# Kinetic and Surface Study of Single-Walled Aluminosilicate Nanotubes and Their Precursors

**DOI:** 10.3390/nano3010126

**Published:** 2013-03-01

**Authors:** Nicolás Arancibia-Miranda, Mauricio Escudey, Mauricio Molina, María Teresa García-González

**Affiliations:** 1 Center for the Development of Nanoscience and Nanotechnology (CEDENNA), Santiago 9170124, Chile; E-Mail: mauricio.escudey@usach.cl; 2 Faculty of Chemistry and Biology, University of Santiago of Chile, Av. B. O’Higgins, Santiago 3363, Chile; 3 Departament of Industry, Federico Santa María Technical University, Av. Santa María 6400, Santiago 766-0251, Chile; E-Mail: mauricio.molina@usm.cl; 4 Institute for Agricultural Sciences, Spanish National Research Council, Serrano 115-dup., Madrid 28006, Spain; E-Mail: mtgg@ccma.csic.es

**Keywords:** metal oxide nanotubes, imogolite, growth kinetics, isoelectric point, electrophoretic characterisation

## Abstract

The structural and surface changes undergone by the different precursors that are produced during the synthesis of imogolite are reported. The surface changes that occur during the synthesis of imogolite were determined by electrophoretic migration (EM) measurements, which enabled the identification of the time at which the critical precursor of the nanoparticles was generated. A critical parameter for understanding the evolution of these precursors is the isoelectric point (IEP), of which variation revealed that the precursors modify the number of active ≡Al-OH and ≡Si-OH sites during the formation of imogolite. We also found that the IEP is displaced to a higher pH level as a consequence of the surface differentiation that occurs during the synthesis. At the same time, we established that the pH of the reaction (pH_rx_) decreases with the evolution and condensation of the precursors during aging. Integration of all of the obtained results related to the structural and surface properties allows an overall understanding of the different processes that occur and the products that are formed during the synthesis of imogolite.

## 1. Introduction

Two low-range-ordered aluminosilicates can be found in volcanic ash-derived soils, one of which consists of single-walled aluminosilicate nanotubes or metal-oxide nanotubes, and is known as imogolite [1]. Its stoichiometry is (OH)_3_Al_2_O_3_SiOH, and it is characterized by a hollow cylindrical structure with an average outer diameter of 2.5 nm and with a length that varies from 100 nm to several micrometres [2,3]. The simplicity of its synthesis and its high chemical flexibility [4,5], which results from the surface groups that constitute and functionalize the imogolite in a natural way (silanols, ≡Si-OH, on the inner surface and aluminols, ≡Al-OH, on the outer surface), have rendered this material an excellent substrate for various nanotechnological applications [6,7,8].

The low yield is the main limitation of the different synthetic methods [5,9,10]. The research conducted to elucidate the mechanism of formation of imogolite has revealed that the subnanometric precursors are formed in the coprecipitation stage and are the key to inducing a self-assembly process during aging [11,12,13,14]. Studies on the mechanism of formation of imogolite, proposed in the literature, agree that the curved structure is due to a series of substitutions of the surface –OH groups of the ≡Al-OH groups of the gibbsite layer with tetrahedral O_3_SiOH. The substitutions result in a curved structure because of the different lengths of the Al-O and Si-O bonds (0.19 nm and 0.16 nm, respectively) [1,2]. A direct consequence of these substitutions is the differentiation of the surface active sites in the precursors, one of which is enriched in ≡Al-OH groups and the other in ≡Si-OH groups.

The synthesis of imogolite in natural systems, or under controlled laboratory conditions, is affected by two key factors: the concentration of the reagents in solution at the beginning of the synthesis (which must have an Al/Si ratio of two, and must be present in concentrations on the order of mM) [15,16] and the temperature (which determines the formation kinetics of the nanoparticles) [17]. These factors play an important role in the growth, dissolution, aggregation, and aging of imogolite.

The formation and characterisation of imogolite has been studied by techniques such as FTIR spectroscopy, X-ray diffraction (XRD), transmission electron microscopy (TEM), X-ray absorption spectroscopy (XAS), nuclear magnetic resonance (NMR), electrospray ionisation mass spectrometry (ESI-MS), and small-angle X-ray scattering (SAXS) [11,18,19]. However, the experimental conditions of the synthesis and the particular limitations of each characterization technique have hindered the determination of an adequate growth mechanism for this aluminosilicate. Research on structures analogous to imogolite, such as aluminogermanates ((OH)_3_Al_2_O_3_GeOH), have led to significant progress toward a general mechanism that explains the formation of imogolite [18,19,20].

Independent of the specific mechanism through which imogolite is obtained [18,19], the magnitude of the structural changes and the marked differences in the chemical reactivity of the groups that are formed during the aging process (≡Al_2_-OH, ≡Al-OH and ≡Si-OH) allow the use of surface characterisation techniques, such as electrophoretic migration (EM). The high sensitivity of the EM technique to small surface changes [20,21,22] allows the evolution of the precursors and their later assembly and growth to be followed.

Electrophoretic migration is a surface characterisation technique that has been widely used to study the surface of catalysts, ore flotation, and pedology, because it allows a good description of the phenomena that occur on a surface [23,24,25,26,27]. The isoelectric point (IEP) of a sample can be determined through EM measurements. This parameter corresponds to the pH at which the net charge of the diffuse layer (σD) is equal to zero and can be used as a descriptive parameter of the surface changes that affect a particle, such as surface enrichment, coating, and hydration [21,22,27]. Although the ≡Al-OH/≡Si-OH active sites ratio is important in an aluminosilicate, its surface distribution is even more significant for defining its IEP.

Several authors have reported that the point of zero charge (PZC) varies with the size of a nanoparticle as a consequence of the changes in composition that occur during growth [28,29]. Within this framework, the objective of this research is to evaluate the structural changes that occur during the aging process of imogolite synthesis—from the monomeric precursors to the formation of the nanotubes—based on the changes in the surface ≡Al-OH/≡Si-OH ratio, as reflected in the evolution of the IEP of each precursor and in the changes in the reaction pH that occur during the synthesis, along with other techniques.

## 2. Experimental Section

Reagent-grade solvents were used. The imogolite sample was prepared using tetraethyl orthosilicate (99.995%, Sigma-Aldrich, St. Louis, MO, USA), NaOH (99.996%, Merck, Darmstadt, Germany), AlCl_3_ (99.998%, Merck), HCl (99.998%, Merck), CH_3_COOH (99.998%, Merck) and NH_4_OH (99.997%, Merck).

*Synthesis of Imogolite.* Tetraethyl orthosilicate (TEOS) was added dropwise to a stirred 5 mM solution of AlCl_3_ solution until the Al:Si ratio was 1.8, and the mixture was allowed to stand for 45 min under vigorous stirring. A NaOH (10^−^^1^ M) solution was subsequently added at a rate of 0.3 mL/min until the pH of the solution was 5.0. The pH was immediately decreased to 4.5 by the dropwise addition of HCl (10^−^^1^ M) and CH_3_COOH (2 × 10^−^^1^ M) solutions. The resulting clear solution was stirred for 3 h and then reacted at 95 °C under reflux. The remaining sample was transferred to a vessel under vigorous stirring. A 0.1 M ammonia solution was carefully added until the pH was 8.0. The solids were concentrated and washed with deionized water by centrifugation at 9000 rpm for 30 min until the specific conductivity was reduced to less than 0.78 dS/m [13].

*Electrophoretic Measurements and Isoelectric Point (IEP)*. The samples were suspended in 200 mL of KCl (1 × 10^−3^ M). The pH was adjusted with HCl or KOH (1 × 10^−2^ M), and electrophoretic mobility measurements were performed on a Zetameter apparatus. The IEP was obtained from the plot of electrophoretic mobility *vs.* pH.

*Point of Zero Salt Effect (PZSE). *The surface charge characteristics of the aluminosilicate samples were measured by potentiometric titration. Aluminosilicate suspensions were prepared by mixing 300 mg of solid samples with 100 mL of KCl at different ionic strengths (10^−1^, 10^−2^ and 10^−3^ M). The titrations were performed under an N_2_ atmosphere at a constant temperature of 25 °C. The titrations were initiated at the original pH, and 0.2 mL of KOH or HCl (10^−1^ M) was added every 20 min. The pH response of the electrode was calibrated with buffer solutions at pH 4.00, 7.00 and 10.00. The PZSE was determined by locating the common intersection point of the potentiometric titration curves at different ionic strengths.

*Transmission Electron Microscopy (TEM)*. The samples were observed under a Zeiss EM 910 transmission electron microscope (Zeiss, Oberkochen, Germany) using an acceleration potential 80 kV. The samples were mounted onto carbon substrates prepared as follows: a drop of the sample, suspended in water, was transferred to the face of a freshly cleaved sheet of mica, and the solvent was allowed to evaporate. A thin layer of carbon was deposited onto the surface by vacuum evaporation. The carbon/product film was separated from the mica sheet by flotation on distilled water and was subsequently transferred to a perforated Cu support grid. Each sample was examined on a LEO 910 transmission electron microscope (Zeiss, Oberkochen, Germany) operated at 120 kV.

*X-Ray Diffraction (XRD)*. XRD analyses were performed using oriented aggregates prepared by drying water suspensions of the samples on glass slides. Samples were scanned from 3° to 70° 2θ using a step size of 0.02° 2θ and scanning for 1.0 s at each step. The X-ray patterns were collected using CuKα radiation from a Philips X’Pert diffractometer generator (Philips, Amsterdam, the Netherlands) and a theta/theta goniometer equipped with a 1.5° divergence slit a 0.2° receiving slit, a graphite diffracted-beam monochromator, and a scintillation counter.

*Fourier-Transform Infrared Spectroscopy (FTIR)*. FTIR spectra of both aluminosilicate compounds were obtained on a Bruker Tensor 27 spectrometer (Bruker, Billerica, MA, USA). A 3-mg dry sample was compacted in a spectral-grade KBr matrix. The spectra were scanned 32 times at a resolution of 2 cm^−1^.

*Specific Surface Area (SSA)*. The SSA and the micropore and mesopore volumes and diameters were determined from nitrogen adsorption–desorption isotherms measured on a Micromeritics model ASAP 2010 (Micromeritics, Norcross, GA, USA) and in a Carlo Erba Sorptmatic 900 (Carlo Erba, Milan, Italy) using the static volumetric method. Samples of 0.500 g were degassed at 483 K for 24 h, with a residual vacuum of 0.532 Pa. The SSA was calculated from the nitrogen adsorption isotherm at 77 K by the Brunauer–Emmett–Teller (BET) method (11). The micropore volume was calculated from the nitrogen adsorption at 77 K by the *t*-plot method; the volume and diameter of the mesopores were calculated from the adsorption/desorption nitrogen isotherm by the Barrett–Joyner–Halenda (BJH) method (11). All samples were analyzed in triplicate.

*Data Analysis. *A variance analysis was performed to compare the IEP and reaction pH (pH_rx_) values at different aging times. The mean values were compared using the Tukey test (*p* < 0.01), which is a conservative test based on the distribution of statistic *q* that allows multiple comparisons and corrects for the increasing risk of finding differences by chance (type I error).

## 3. Results and Discussion

### 3.1. Structural Characterisation

**Figure 1:**
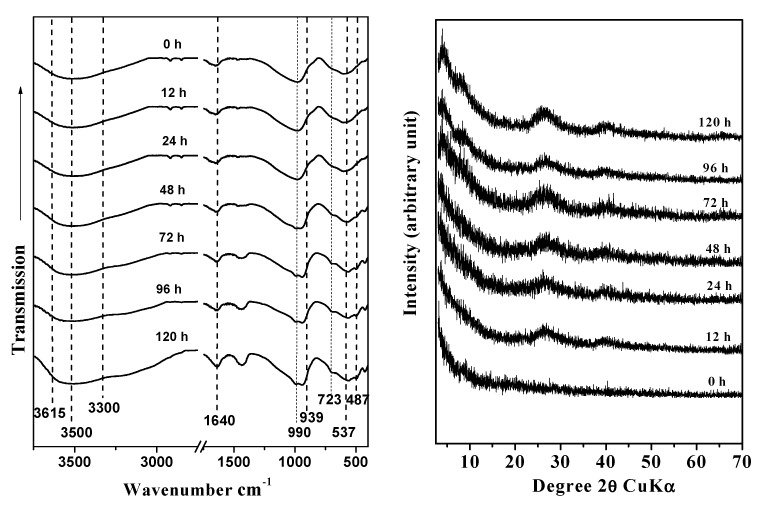
Fourier-Transform Infrared Spectroscopy (FTIR) spectra (**a**) and X-Ray Diffraction (XRD) patterns (**b**) of the synthetic products at different aging times: (**a**) 0 h, (**b**) 12 h, (**c**) 24 h, (**d**) 48 h, (**e**) 72 h, (**f**) 96 h, and (**g**) 120 h.

The morphological changes of imogolite during the aging process were analyzed by FTIR spectroscopy, XRD, and TEM. The FTIR spectra collected at different preparation times showed the presence of three wide peaks at 1080, 968, and 590 cm^−1^, which correspond to the stretching of the Si-O and Si-O-Al bonds and are consistent with the structure of proto-imogolite (48 h). After 72 h of aging, bands appeared at 990 and 939 cm^−1^, which belong to the Si-O stretching vibrations that are specific to tubular structures; these bands remained almost invariable throughout the aging time (Figure 1a) [5,30,31,32]. Changes in hydroxide bands were also observed in the 3500–3200 cm^−1^ range. After 72 of aging, –OH bands at 3300 and 3500 cm^−1^ associated with external ≡AlOH and ≡Al_2_OH were observed; in the same spectrum, a band at 3615 cm^−1^ associated with ≡Si-OH sites located in the inner surface of ring-type precursors was present (Figure 1a) [33]. 

The evolution of the diffraction patterns of samples obtained at different aging times are summarized in Figure 1b. The XRD data show that the precursor formed after the coprecipitation process had ended corresponds to an amorphous aluminosilicate [34]. The precursor remained for the first 48 h of aging. We observed a better definition of the diffraction patterns of the precursors formed was observed as the aging progressed, as indicated by wide diffraction patterns at 72 h of aging, corresponding to 0.21, 1.20, 0.34 and 0.22 nm. These bands, which are characteristic of a paracrystalline structure, such as that of imogolite, remained almost invariable until the end of the aging process; the bands became more defined with time, which we interpreted as a sign of the extension of the aging process [14,34,35].

**Figure 2:**
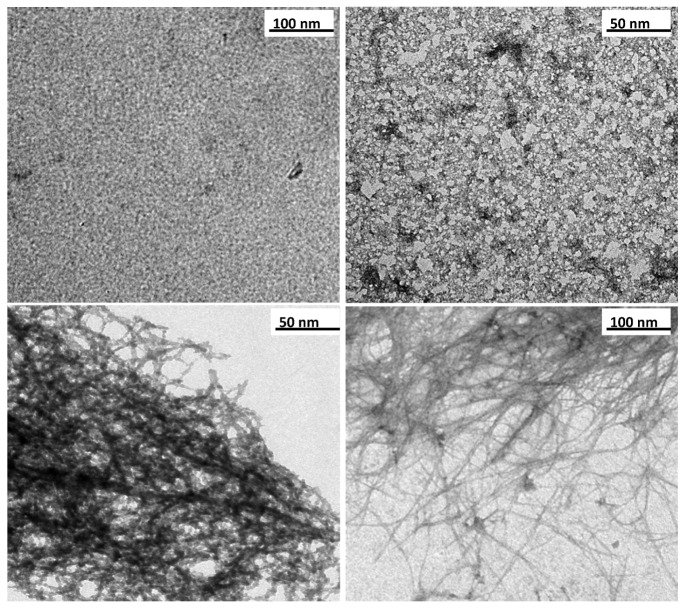
Transmission Electron Microscopy (TEM) images of products at different aging times: (**a**) 0 h, (**b**) 24 h, (**c**) 72 h, and (**d**) 120 h.

The TEM observations made during the first hours of the synthesis show that the precursors formed have an ill-defined and short-range structure under X-ray and that they reached a size of approximately 5 nm (Figure 2a).

As a consequence of aging and the increasing substitution of the –OH of the ≡Al-OH groups by O_3_SiOH, the precursors start growing and forming complex oligomers with sizes smaller than 15 nm (Figure 2b). The TEM results indicate that the maximum structuring of the precursors was achieved between 48 and 72 h after the beginning of the aging process; the images show the condensation of nanotube pieces approximately 20 to 30 nm in size (Figure 2c), which evolve into the structure characteristic of imogolite (Figure 2d).

Based on nitrogen adsorption measurements, the textural properties of the products obtained at different aging times were determined (Table 1).

During the aging process, the BET surface area increases as a result of changes in the spatial arrangement and growth of the precursors (Figure 2) [36]. With the exception of SSA, after 72 h of aging, the textural properties of the precursors were similar to those of the end product (imogolite). These results are consistent with the XRD, FTIR and TEM data. During the aging process, under the experimental conditions considered, the SSA increased even after 120 h, reaching 351 m^2^·g^−1^ at 240 h.

**Table 1:** Textural features of products obtained at different aging times.

Time (h)	Surface area ^a^ (m^2^/g)	Pore volume ^b^ (cm^3^/g)	Microporous volume ^c^ (cm^3^/g)	Inner diameter (nm)	Outer diameter (nm)
0	170	0.10	0.04	---	---
12	181	0.13	0.03	---	---
24	197	0.15	0.03	---	---
72	214	0.20	0.02	1.0	2.3
96	253	0.23	0.02	1.0	2.5
120	303	0.25	0.02	1.0	2.5

Notes: ^a^ Specific surface area, as calculated according to the BET (Brunauer−Emmett−Teller) method through multipoint calculation by choosing the result given by the best linear-fit in the 0.1 to 0.2 *P*/*P*^0^ range; the resulting estimated error is 3% of the obtained value. ^b^ Pore volume was obtained from the N_2_ adsorption isotherm performed at 77 K. ^c^ As derived by applying the α_s_ method.

### 3.2. Surface Characterization

The IEP is a parameter that indicates the level of enrichment in ≡Si-OH and ≡Al-OH groups of the new surfaces that result from the aging process, and its value changes until it reaches the characteristic IEP of imogolite [24,36,37,38].

**Figure 3:**
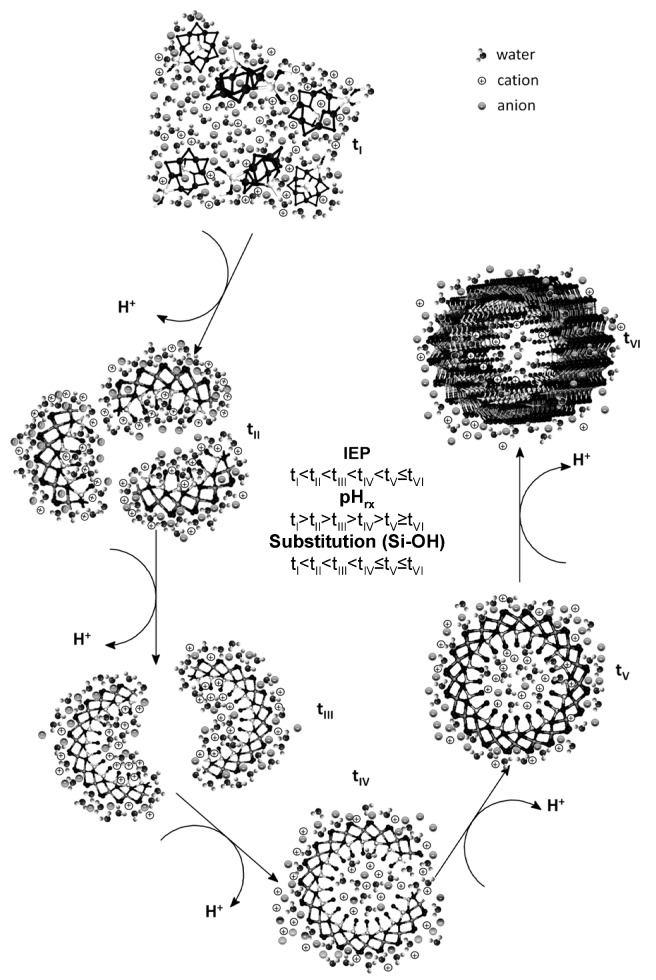
Structural evolution of the precursors formed during the synthesis of imogolite.

The variation of the IEP as a function of time is summarized in Table 2. The IEP obtained for the precursor formed after the coprecipitation of the starting products had ended suggests that the numbers of ≡Si-OH, ≡Al-OH and ≡Al_2_-OH groups are similar and that their distribution on the surface is heterogeneous (Table 2) (t_I_→t_II_, Figure 3).

**Table 2:** Isoelectric point (IEP), reaction pH (pH_rx_), ∆IEP/∆t, and ∆pH_rx_/∆t as a function of the synthesis time.

Time (h)	IEP	∆IEP/∆t	pH_rx_	∆pH_rx_/∆t
0	6.6 a	0	4.49 a	0
12	7.1 b	4.2	4.26 b	1.90
24	7.8 c	5.8	3.85 c	3.40
48	8.5 d	2.9	3.61 d	1.80
72	10.0 e	6.3	2.78 e	3.50
96	10.3 ef	1.3	2.72 f	0.03
120	10.6 fg	1.3	2.70 fg	0.01

Note: Within columns, values followed by the same letter are not significantly different according to Tukey’s test (*p* < 0.01). The ∆IEP/∆t and ∆pH_rx_/∆t values are amplified by a factor of 10.

As the self-assembly of the precursors occurs, they evolve into more complex structures, and their IEPs shift to more basic pH values. This evolution is the result of the increased substitution of –OH groups from ≡Al-OH by O_3_SiOH during aging (self-assembly), which causes the ≡Al-OH, ≡Al_2_-OH and ≡Si-OH groups to become localized on separate surfaces and the ≡Al-OH, ≡Al_2_-OH groups to become exposed as outer active sites (t_II_→t_III_→t_IV_, Figure 3) [38,39,40].

Between 48 and 72 h of aging, the substitutions are most significant, which results in some of the formed precursors to be completely closed (*i.e.*, to form ring structures). The surface characteristics of these types of precursors would account for the increase in the IEP by 1.5 pH units because the outer surface has a clear predominance of ≡Al-OH and ≡Al_2_-OH groups (t_V_, Figure 3) [2,38,40].

After the ring structure has formed, the IEP variation is minimal because the ≡Si-OH groups are found preferentially on the inner surface of the precursor, which decreases their contribution to the surface charge of the imogolite (t_V_, Figure 3) observed by electrophoretic migration. Toward the end of the synthesis (96–120 h), the IEP values obtained are close to those reported for gibbsite (10.6–11.0), a main constituent of the outer surface of imogolite (t_VI_, Figure 3) [35,36,37,38,39,40,41]. The IEP increased after 120 h and reached 10.7, which, according to the Tukey test, was not a significant change. To understand the IEP values and variations observed in the precursors and in imogolite, it is important to consider that, in pure silicon and aluminium oxides, this parameter assumes values of approximately two and nine, respectively [39] and that, in binary compounds such as allophane (a short-range-ordered aluminosilicate), an IEP lower than that of imogolite has been determined, despite both having a similar Al/Si ratio [34]. This result is explained by the close relationship between the structural and surface arrangement of the ≡Si-OH and ≡Al-OH groups and the IEP value.

**Figure 4:**
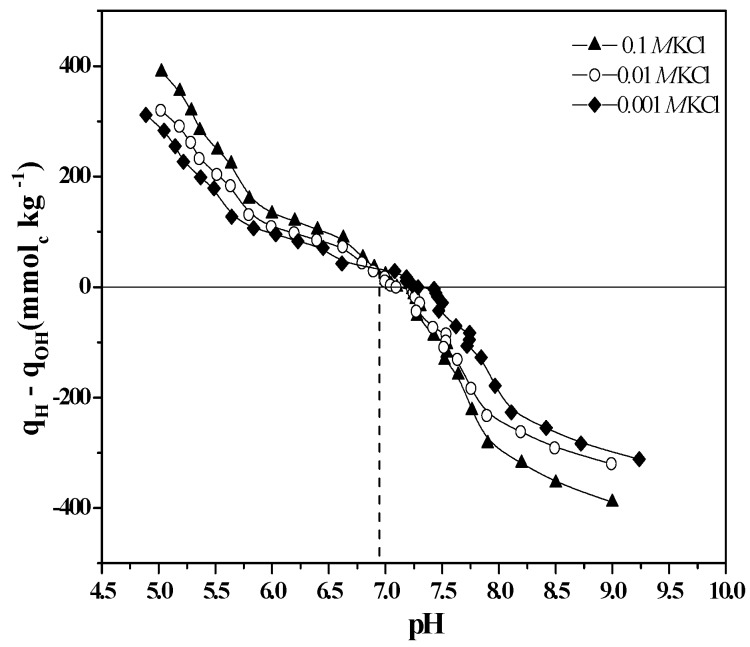
Potentiometric titration curves of imogolite at three different KCl concentrations.

The contribution of ≡Si-OH groups to EM measurements decreases as the imogolite grows because the exposed surface contains mostly ≡Al_2_-OH and ≡Al-OH sites, which prevents the determination of the contribution of the ≡Si-OH groups at the charge level. Through a comparison of the IEP (determined by electrokinetic measurements) and the PZSE (determined by potentiometric measurements), the issue of whether silanol groups participate in ion-exchange reactions after the formation of imogolite nanotubes can be determined (Figure 4). The IEP and PZSE pH values agree only when the active surface sites that participate in charge generation are the same [27].

An IEP of 10.5 was determined for imogolite, which shows the influence of the ≡Al_2_-OH and ≡Al-OH groups that dominate the outer surface composition of the nanotubes [2,34,38,39,40]. In contrast, the PZSE of 6.8 is the result of proton exchange reactions that occur on both internal ≡Si-OH and external ≡Al-OH active sites [27,34].

The analyses of the IEP and PZSE indicate that the surface changes drastically and shows that the outer surface Al/Si ratio increases under the effect of the aging process. This phenomenon conditions the arrangement of the imogolite reactive groups and explains the differences in the observed IEP and PZSE values; these differences are attributable to the presence of two different types of functional groups dominating the inner and outer surfaces of the tubular structure of the nanoparticle.

**Figure 5:**
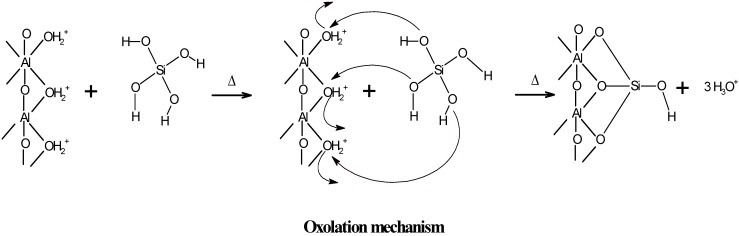
Schematic representation of the substitutions of –OH groups of the Al precursors by the orthosilicate anion.

These results confirm that the active sites of the outer surface of imogolite are Al-OH enriched and that the ≡Si-OH groups located on the inner surface of the imogolite nanotube are also part of the reactive groups during the potentiometric titration (Figure 4) [4,27,40]. During aging, another synthesis parameter, the pH of reaction (pH_rx_), decreases due to condensation of the precursors, which occurs through an oxolation mechanism (Figure 5)—a process that releases H_3_O^+^ into the solution [12,16,30,38].

**Figure 6:**
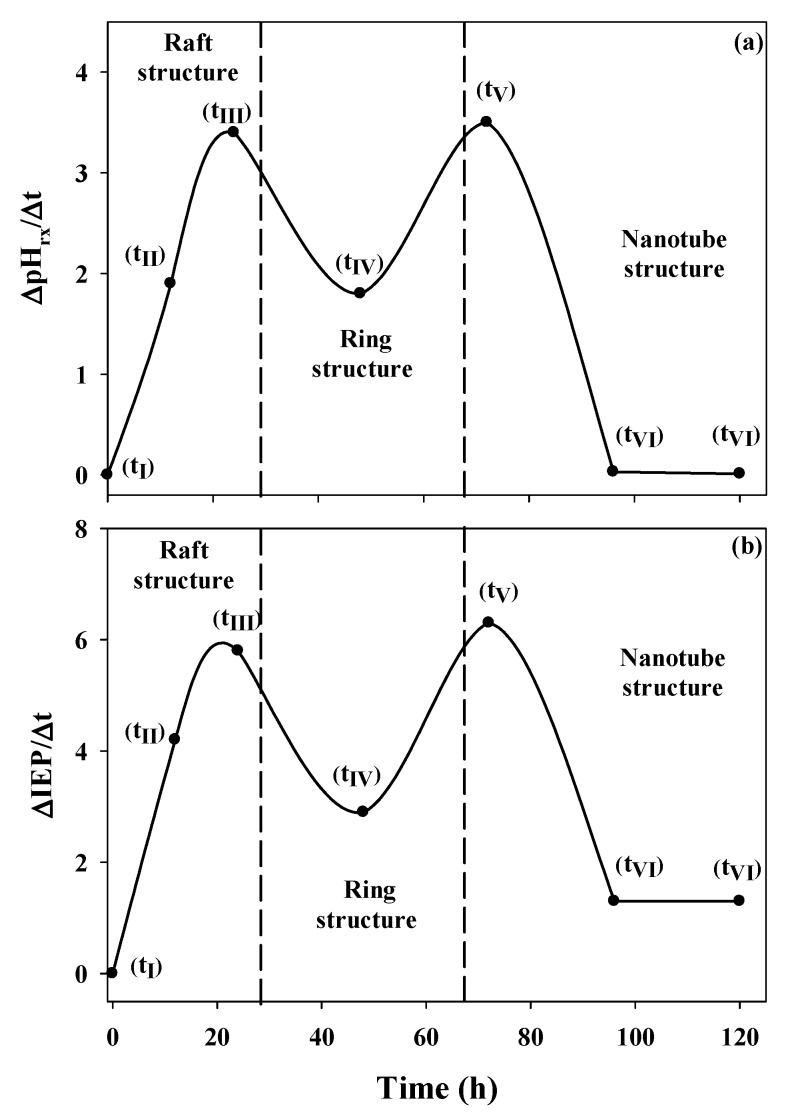
Changes in the reaction pH (∆pH_rx_/Δt) and the isoelectric point (∆IEP/Δt) as functions of the aging time.

The variation of the pH of reaction is similar to that of the IEP during the aging process within the same time interval, which shows that both parameters are dependent on the structural changes to which the precursors are subjected during aging. A more significant way of observing the effect of structural changes on the pH_rx_ is to represent the variation of this parameter as a function of aging time (Table 2, Figure 6a).

This graph clearly shows that the most significant structural changes result in the most important changes in pH_rx_. Three important changes occur: the first is the change from the monomeric and oligomeric precursors (t_I_, Figure 3) to the raft-type structure (t_III_, Figure 3); the second change corresponds to the path from the raft-type structures to rings (t_III_→t_V_, Figure 3); and the third change accounts for the one-dimensional growth of the ring-type structure until it forms the imogolite nanotube (t_V_→t_VI_, Figure 3). A similar association can be made when the change in the IEP as a function of the change in time (∆IEP/∆t) is evaluated (Figure 6b). This fact shows that both parameters are dependent on the structural changes to which the precursors are subjected during aging. This strong relationship between ∆IEP/∆t and ∆pH_rx_/∆t was confirmed through a correlation analysis (*r* = 0.9492, *p* = 0.0002).

### 3.3. Surface Evidence of the Formation of Imogolite

The combined use of different analytical techniques (FTIR, XRD, TEM and EM) allowed the identification of two key stages that occur during aging in the synthesis of imogolite, which are described by the mechanisms of formation of imogolite proposed by Maillet *et al.* and Yucelen *et al.* [18,19].

The first stage is the formation of precursors that will evolve into imogolite, and, according to Maillet *et al.*, they correspond to short-range amorphous subnanometric species. However, according to Yucelen *et al*., the precursors formed are of the Keggin-ion type, and both precursors originate from the hydrolysis of the starting reagents [18,19].

The growth and development of these precursors into more complex structures (*i.e.*, raft and ring structures) occurs through a self-assembly mechanism that originates during Ostwald ripening [42,43,44], a process associated with reactions that involve thermal aging, such as the case of imogolite synthesis [11,14,16,19]. These processes are those that produce the most significant changes in the precursors at the structural and surface level, as indicated by the IEP and pH_rx_ values (Figure 5).

However, with respect to the growth of the imogolite tubes (*i.e.*, the second stage), both mechanistic models differ on the route that converts the annular precursors into nanotubes. According to Maillet *et al.*, the formation of imogolite occurs as a consequence of successive collisions of the nanotube fragments (*i.e.*, tip–tip collisions) [18]. If the growth process of imogolite occurs along this route, the electrokinetic behavior of the precursors, which is reflected in the IEP values, should be similar starting from 48 h of aging until the end of the aging process. This similarity occurs because the precursors formed in this time have a surface with similar active sites of the imogolite nanotubes, which is not what was determined from the electrophoretic measurements (Table 2).

Our experimental evidence of the surface behavior of the different precursors obtained during the synthesis of imogolite suggests that its growth is governed by dissolution and condensation processes of those precursors that are in thermodynamically less stable states; the precursors rearrange themselves until the nanotubes are produced, as proposed by Yucelen *et al.* [19], which accounts for the variations in the IEP and pH_rx_ values observed in this study (Figure 3, Figure 6; Table 2).

The surface differentiation observed in the precursors due to the effect of the substitution of the –OH groups that belong to ≡Al-OH by O_3_SiOH and the one-dimensional growth of the imogolite nanotubes are stages that are highly unfavored from the standpoint of entropy. However, the energy released during both processes, and mainly during the condensation of the precursors (*i.e.*, the oxolation mechanism), contributes to the enthalpy factor, which explains the favorable formation of imogolite [16,44,45], as strongly evidenced by the changes in the surface composition of the precursors.

Our results allow a better understanding of surface reactivity changes during the imogolite aging process. This information is relevant to the design of new nanomaterials based on imogolite. The elaboration of nanocables based on metals such as Cu, Ag, and Au insulated by an imogolite coating [19,28] could be considered in electrical nanodispositives [46]. A Cu@imogolite, Ag@imogolite or Au@imogolite nanocable is possible because the imogolite synthesis conditions (considering the acidic pH conditions during imogolite synthesis) promote the adsorption of cations onto ≡Si-OH surface active groups located in the inner surface of imogolite precursors [4].

## 4. Conclusions

Micrographs taken at different stages of preparation showed the structures formed during the aging process, which evolve from amorphous structures to imogolite nanotubes. The FTIR spectra confirmed the presence of protoimogolite during the first hours of synthesis and the formation of a ring-type structure at 72 h of aging, after which no important changes were observed by FTIR at longer aging times. These results indicate that this technique loses sensitivity after the ring-type precursor is formed.

However, the surface and structural changes, such as the formation, evolution, and growth of the precursors, can be followed until the final structure of imogolite is obtained through the variations in the IEP obtained by EM. This approach allows the interpretation of the surface phenomena that occur during the aging of imogolite (*i.e.*, self-assembly and Ostwald ripening). We established that, during the process of imogolite formation, the precursors present in the solution evolve into different types of structures (*i.e.*, raft, ring and nanotube structures) as a result of the substitutions of -OH of the ≡Al-OH groups by O_3_SiOH. The increase in the IEP values is the consequence of structural and surface changes that occur in the aging process to obtain imogolite, which are due to the observed phenomenon of surface differentiation, where the outer and inner surfaces have different electrophoretic behaviors. The PZSE of imogolite indicated that ≡Al-OH and ≡Si-OH groups are located on clearly separate surfaces in view of the difference observed between the IEP and PZSE values. The condensation of the precursors caused a decrease in the pH_rx_, which was significant between 48 and 72 h of aging—a time during which the predominant structure is the ring type.

Based on our results, the critical stage in the synthesis of imogolite corresponds to the formation of a ring type structure that, from a thermodynamic point of view, most likely led to a globular structure (*i.e.*, an allophane-like structure); however, the high dilution, the Al/Si ratio, and the extended aging period used during the synthesis hindered this transformation, which caused the terminal functional groups—the constituents of the tip of the annular structure (≡Al-OH and ≡Si-OH)—to constantly consume precursors in an attempt to achieve a globular structure. As a consequence, we observed only one-dimensional growth of the precursors, which evolved into imogolite.
